# Supplementary material for: Characterization of Promising Cytotoxic Metabolites from Tabebuia guayacan Hemsl.: Computational Prediction and In Vitro Testing
Source: Plants (Basel). 2022 Mar 26;11(7):888. doi: 10.3390/plants11070888 (PMC9002841; doi:10.3390/plants11070888)
Supplement: Supplementary file 1 [file plants-11-00888-s001.zip › plants-1630872-supplementary.pdf]

plants

MDPI

Article

Characterization of Promising Cytotoxic Metabolites from *Tabebuia guayacan*: Computational Prediction and In Vitro Testing

Seham S. El-Hawary [1], Rabab Mohammed [2], Marwa A. Taher [3], Sameh Fekry AbouZid [2,4], Mostafa A. Mansour [5], Suliman A. Almahmoud [6], Bader Huwaimel [7] and Elham Amin [2,6,*]

Supplementary Materials

1. Compounds identification data

The chromatographic investigation of three different fractions (CH_2Cl_2, EtOAc and *n*-butanol) from the leaves of *T. guayacan* Hemsl. lead to the isolation of sixteen compounds **1-16** (Figure 2).

Compound 1 (β-sitosterol): White needle crystals, m.p. 140-142 ºC, freely soluble in *n*-hexane, positive test for sterol and/or triterpenes, R_f = 0.49, give violet color with *p*-anisaldehde/ H_2SO_4/ heat at 110 ºC, LC-HRMS $[M+H]^+$ m/z: 415.7065, MW: 414.7067, R*t*: 10.21 calculated for $C_{29}H_{50}O$. ^{1}H-NMR (400 MHz, $CDCL_3$): δ 5.35 (1H, *d*, *J*=3.6, H-6), 3.49 (1H, *m*, H-3), 1.02 (3H, *s*, Me-19), 0.93 (3H, *d*, *J*= 4.4, Me-21), 0.86 (3H, *d*, *J*=2.4, Me-26), 0.84 (3H, *d*, *J*=1.6, Me-27), 0.82 (3H, *d*, *J*=2.4, Me-29), 0.69 (3H, *s*, Me-18) ppm. DEPT-Q NMR (100 MHz, $CDCL_3$): δ 37.3 (C-1), 31.9 (C-2), 71.8 (C-3), 42.3 (C-4), 140.8 (C-5), 121.7 (C-6), 31.9 (C-7), 31.9 (C-8), 50.1 (C-9), 36.5 (C-10), 21.1 (C-11), 39.8 (C-12), 42.29 (C-13), 56.8 (C-14), 24.3 (C-15), 28.3 (C-16), 56.1 (C-17), 11.9 (C-18), 19.4 (C-19), 36.1 (C-20), 19.0 (C-21), 33.9 (C-22), 26.1 (C-23), 45.8 (C-24), 29.1 (C-25), 19.8 (C-26), 18.8 (C-27), 23.1(C-28), 11.9 (C-29).

Compound 2 (Ursolic acid): White powder, m.p. 285-288 ºC, soluble in methanol, R_f = 0.53 in system [Methylene chloride: Methanol (90: 10 v/v)], positive test for sterol and/or triterpenes, Purple color with *p*-anisaldehde/ H_2SO_4/ heat at 110 ºC, LC-HRMS $[M+H]^+$ m/z: 457.36726, MW: 456.35998, Rt: 21.25 calculated for $C_{30}H_{48}O_3$. ^{1}H-NMR (400 MHz, CD_3OD): δ 5.24 (1H, *t*, H-12), 3.15 (1H, *dd*, *J* = 11.0, 4.5Hz, H-3), 2.21 (1H, *d*, *J* = 10.5 Hz, H-18), 2.31(1H, *ddd*, *J* = 13.5, 13.0, 4.5 Hz, H-16), 1.97 (1H, *ddd*, *J* = 13.0, 12.5, 3.5 Hz, H-1), 1.18 (3H, *s*, Me-23), 1.14 (3H, *s*, Me-27), 1.0 (3H, *s*, Me-26), 0.97 (3H, *d*, *J* = 6.0 Hz, Me-30), 0.90 (3H, *d*, *J* = 6.5 Hz, Me-29), 0.87 (3H, *s*, Me-24), 0.80 (3H, *s*, Me-25). DEPT-Q (100 MHz, CD_3OD): δ 38.91(C1), 27.35(C-2),78.13(C-3), 39.05 (C-4), 55.239 (C-5), 18.47(C-6), 33.17(C-7),40.11(C-8),47.40(C-9), 36.99 (C-10), 23.19(C-11), 125.05 (C-12), 138.66 (13),42.11 (C-14), 28.01(C-15), 24.27(C-16), 47.30(C-17), 52.85 (C-18), 38,79(C-19), 38.70 (C-20), 30.65 (C-21), 36.79 (C-22), 28.70(C-23), 15.57 (C-25), 16.55(C-26), 23.74(C-27), 179.78 (C-28), 17.3(C-29), 21.60 (C30).

Compound 3 (Corosolic acid): white powder, m.p. 243-245 ºC, soluble in methanol, R_f = 0.55 in system [Methylene chloride: Methanol (90: 10 v/v)], positive test for sterol and/or triterpenes, Purple color with *p*-anisaldehde/ H_2SO_4 / heat at 110 ºC, LC-HRMS [M+H] + m/z: 473.36206, MW: 472.35478, Rt: 19.25 calculated for $C_{30}H_{48}O_4$. ^{1}H-NMR (400 MHz, CD_3OD): δ 5.14 (1H, *t*, H-12), 4.30 (1H, *d*, *J* = 9.5, Hz, H-2), 3.01 (1H, *m*, H-3), 2.10 (1H, *d*, *J* = 11.5 Hz, H-16), 1.95 (1H, *ddd*, *J* = 13.0, 12.5, 4.5 Hz, H-1), 1.10 (3H, *s*, Me-23), 1.05 (3H, *s*, Me-27), 0.90 (3H, *s*, Me-26), 0.86 (3H, *d*, *J* = 6.0 Hz, Me-30), 0.81 (3H, *d*, *J* = 6.5 Hz, Me-29), 0.76 (3H, *s*, Me-24), 0.69 (3H, *s*, Me-25). DEPT-Q (100 MHz, CD_3OD): δ178.84 (C-28), 138.73 (13), 124.93 (C-12), 82.73 (C-3), 67.64 (C-2), 55.19 (C-5), 52.85(C-18). 47.53 (C-9),47.44 (C-17), 47.30 (C-1), 42.16 (C-14), 38.96 (C-8), 38.90 (C-4), 38.10 (C-19), 38.06 (C20), 36.86 (C-10), 36.78 (C-22), 33.08 (C-7), 30.62 (C-21), 29.28 (C-23), 27.94 (C-15), 24.26 (C-16), 23.72 (C-27), 23.39 (C-11), 21.53(C-29), 18.47(C-6), 17.62(C24), 17.45(C26), 16.87(C-30),16.74(C-25).

Compound 4 (3-*O*-*p*-coumaroyl corosolic acid): white powder, m.p. 265 ºC, soluble in methanol, R_f = 0.58 in system [Methylene chloride: Methanol (90: 10 v/v)], positive test for sterol and/or triterpenes, Purple color with *p*-anisaldehde/ H_2SO_4 / heat at 110 ºC, LC-HRMS [M+H] + m/z: 619.3984, MW: 618.39112, R*t*: 24.91 calculated for $C_{39}H_{54}O_6$. ^{1}H-NMR (400 MHz, CD_3OD): δ 7.67 (1H, *dd*, *J* = 8.7, 15.9 Hz, H-7'), δ 7.48 (2H, *d*, *J* = 8.6 Hz, H-2' and H-6'), δ 6.77 (2H, *ddd*, *J* = 8.6, 9.9, 15.6 Hz, H-3' and H-5'), δ 6.39 (1H, *d*, *J* = 15.9 Hz, H-8'), δ 5.86 (1H, *d*, *J* = 9.9 Hz, H-3), δ 5.27 (1H, *br s*, H-12), 4.61 (1H, *dd*, *J* = 11.0, 4.5Hz, H-2), 2.02 (1H, *d*, *J* = 10.5 Hz,H-18), 2.31(1H, *ddd*, *J* = 13.5, 13.0, 4.5 Hz, H-16), 2.13 (1H, *ddd*, *J* = 13.0, 12.5, 3.5 Hz, H-1), 1.21 (3H, *s*, Me-23), 1.16 (3H, *s*, Me-27), 1.12 (3H, *s*, Me-26), 1.0 (3H, *d*, *J* = 6.0 Hz,Me-30), 0.9 (3H, *d*, *J* = 6.5 Hz, Me-29), 0.85 (3H, *s*, Me-25), 0.88 (3H,*s*, Me-24). DEPT-Q (100 MHz, CD_3OD): δ179.28 (C-28), 1681.92 (C-9'), 159.77(C-4'), 144.89 (C-8'), 138.40 (13), 129.75 (C-2' and 6'), 125.94 (C-1'), 125.20 (C-12), 115.46 (C-5'), 114.45 (C-3'), 84.16(C-3), 66.25(C-2), 55.05(C-5), 52.94(C-18). 48.27(C-1), 48.05(C-9), 47.84(C-17), 41.39(C-14), 39,45(C-8), 39.26(C-4), 39.01(C19-20), 37.81(C-10), 36.70(C-22), 32.74(C-7), 32.42(C-21), 30.37(C-23),27.91(C-15), 23.91(C-16), 23.07(C-27), 22.79(C-11), 20.22(C-29), 18.66(C-6), 17.04(C26), 16.41(C-30), 16.29(C-25).

Compound 5 (3β-6β-19α-trihydroxy-urs-12-en-28-oic acid): White powder, m.p. 280 ºC, soluble in methanol. R_f = 0.65 in system [Methylene chloride: Methanol (90: 10 v/v)], positive test for sterol and/or triterpenes. Purple to violet color with *p*-anisaldehde/ H_2SO_4 / heat at 110 ºC, LC-HRMS [M+H] + m/z: 489.35693, MW: 488.34965, R*t*: 15.84 calculated for $C_{30}H_{48}O_5$. ^{1}H-NMR (400 MHz, $CDCL_3$): δ 5.32 (1H, *br t*, H-12), 4.51 (1H, *m*, H-6), 3.54 (1H, *d*, H-3), 2.06 (1H, *d*, *J* = 11.5 Hz, H-18), 2.55(1H, *m*, H-16), 2.29 (1H, *m*, H-11), 1.85 (3H, *s*, H-25), 1.69 (3H, *s*, H-24), 1.64 (3H, *s*, H-29), 1.51 (3H, *s*, H-26), 1.27 (3H, *s*, H-23), 0.87 (3H, *d*, *J* = 6.5 Hz, H-30). DEPT-Q (100 MHz, $CDCL_3$): δ 42.05(C-1), 26.47(C-2), 79.77(C-3), 39.56(C-4), 56.01(C-5), 71.72(C-6), 40.30(C-7), 38,67(C-8), 47.36(C-9), 36.91(C-10), 23.64(C-11), 129.55 (C-12), 137.80 (13), 41.58(C-14), 28.31(C-15), 25.51(C-16), 47.81(C-17), 53.34 (C-18), 73.46(C-19), 41.16 (C 20), 26.08(C-21), 37.49(C-22), 16.74(C-23), 28.02(C24), 16.66(C-25), 18.34(C-26), 24.43(C-27), 182.73 (C-28), 27.06(C-29), 16.11(C30).

Compound 6 (β-sitosterol-3-*O*-β-D-glucoside) (Daucosterol): white amorphous powder, m.p. 290 ºC, freely soluble in a mixture of methylene chloride and methanol, R_f =0.36 in system [Methylene chloride: Methanol (95: 5 v/v)] and R_f = 0.58 in system [Methylene chloride: Methanol (90: 10 v/v)], Purple color with p-anisaldehde/ H_2SO_4 / heat at 110 ºC, It gave positive Libermann-Burchard's test indicating it's steroidal or triterpenoidal nature and gave positive with Molish's test indicating its glycosidic nature, LC-HRMS [M+H]+ m/z: 577.24627, MW: 576.2389, Rt: 11.96 calculated for $C_{35}H_{60}O_6$. ^{1}H-NMR (400 MHz, DMSO): δ5.34 (1H, *t*, H-6), 3.56 (1H, *m*, H-3), 0.96 (3H, *s*, Me-19), 0.91 (3H, *d*, *J*= 5.6 Hz, Me-21), 0.84 (3H, *d*, *J*= 8.0 Hz, Me-27), 0.82 (3H, *d*, *J*= 7.2 Hz, Me-26), 0.79 (3H, *t*, Me-29), 0.66 (3H, *s*, Me-18), glucose moiety; 4.22 (1H, *d*, *J*= 7.8 Hz, H-1`), 3.13 -3.83 (glc., *m*, 5H). DEPT-Q (100 MHz, DMSO): δ 37.31 (C-1), 29.80 (C-2), 77.42 (C-3), 39.72 (C-4), 140.92 (C-5),121.69 (C-6), 31.90 (C-7), 31.89 (C-8), 50.1 (C-9), 36.68 (C-10), 21.10 (C-11), 38.92 (C-12), 42.33 (C-13), 56.66 (C-14), 24.34 (C-15), 28.28 (C-16), 55.90 (C-17), 12.25 (C-18), 19.10 (C-19), 35.96 (C-20), 19.41 (C-21),

33.82 (C-22), 25.98 (C-23), 45.61 (C-24), 29.17 (C-25), 19.65 (C-26), 20.20 (C-27), 23.1 (C-28), 12.14 (C-29), glucose moiety 101.25 (C-1'), 73.83 (C-2'), 77.23 (C-3'), 70.55 (C-4'), 77.20 (C-5'), 61.71 (C-6').

Compound 7 (Quercetin): yellow powder, m.p. 316 °C, soluble in methanol, R_f = 0.55, (methylene chloride: methanol, 9:1) on silica gel sheets, yellow color with *p*-anisaldehde/ H_2SO_4 / heat at 110 ºC, yellow color in $UV_{365\ nm}$, yellow color with UV/NH_3 and the color is intensified with UV/$AlCl_3$, LC-HRMS $[M+H]^+$ m/z: 303.0498, MW: 302.0425, R*t*: 14.52 calculated for $C_{15}H_{10}O_7$. ^{1}H-NMR (400 MHz, CD_3OD): δ 6.19 (1H, *d, J*=2.0, H-6), 6.39 (1H, *d, J*=2.0, H-8), 6.89 (1H, *d, J*=8.4, H-5'), 7.63 (1H, *dd, J*=1.6, 8.4 H-6'), 7.75 (1H, *d, J*=1.6, H-2'). DEPT-Q (100 MHz, CD_3OD): 147.34 (C-2), 135.12 (C-3), 175.93 (C-4), 161.02 (C-5), 97.85 (C-6), 164.19 (C-7), 93.02 (C-8), 156.81 (C-9),103.09 (C-10), 122.76 (C-1'), 114.58 (C-2'), 144.79 (C-3'), 146.58 (C-4'), 114.82 (C-5'), 120.27 (C-6').

Compound 8 (Luteolin): Light yellow powder, m.p. 338-330 ºC, freely soluble in methanol, R_f = 0.48 (chloroform: methanol, 9:1), on silica gel sheets, yellow color with *p*-anisaldehyde/ H_2SO_4 / heat at 110 ºC, purple color in UV365 nm, yellow color with UV/NH_3 and the color is intensified with UV/$AlCl_3$, LC-HRMS $[M+H]^+$ m/z: 287.05484, MW: 286.04575, R*t*: 15.91 calculated for $C_{15}H_{10}O_6$. ^{1}H-NMR (400 MHz, CD_3OD): δ 7.39 (2H, *d, J*=6.4 Hz, H-2', H-6'), 6.91 (1H, *d, J*=8.8Hz, H-5'), 6.55 (1H, *s*, H-3), 6.45 (1H, *d, J*=1.6 Hz, H-8), 6.22 (1H, *d, J*=1.2Hz, H-6). DEPT-Q (100 MHz, CD_3OD): δ 164.35 (C-2),103.52 (C-3), 182.2 (C-4), 162.01 (C-5), 99.34(C-6), 164.84 (C-7), 94.30 (C-8), 157.97 (C-9), 104.32 (C-10), 119.15 (C-1'), 114.16 (C-2'), 146.11 (C-3'), 150.08 (C-4'), 116.33 (C-5'), 122.26 (C-6').

Compound 9 (Quercetin-3-*O*-β-D-glucoside (Isoquercitrin)): yellow powder, m.p. 226 ºC, soluble in methanol, positive Molisch's test (glycosidic nature), R_f = 0.53, (methylene chloride: methanol, 8:2) on silica gel sheets, dark yellow color with p-anisaldehde/ H_2SO_4 / heat at 110 ºC, yellow color in UV365 nm, yellow color with UV/NH_3 and the color is intensified with UV/$AlCl_3$. LC-HRMS $[M+H]^+$ m/z: 465.10244, Mw: 464.09517, R*t*: 11.05 calculated for $C_{21}H_{20}O_{12}$. ^{1}H-NMR (400 MHz, CD_3OD): Aglycone part: δ 6.20 (1H, *d, J*=2.0, H-6), 6.39 (1H, *d, J*=2.0, H-8), 6.87 (1H, *d, J*=8.4, H-5'), 7.58 (1H, *dd, J*=1.8, 8.4 H-6'), 7.86 (1H, *d, J*=1.6, H-2'), Sugar moiety: δ 5.15 (1H, *d, J*=7.2 Hz, H-1''), 3.88 (1H, *dd, J*=11.4; 5.0 Hz, H-5''b), 3.46-3.54 (2H, *m*, H-3'', H-4''), 3.39 (1H, *m*, H-2''), 3.1 (1H, *dd, J*=11.5; 9.5Hz, H-5''a). DEPT-Q (100 MHz, CD_3OD): A glycone part: δ 157.15 (C-2), 134.43 (C-3), 178.13 (C-4), 161.87 (C-5), 98.85 (C-6), 164.99 (C-7), 93.55 (C-8), 157.54 (C-9), 104.13 (C-10), 121.76 (C-1'), 114.73 (C-2'), 148.78 (C-3'), 144.64 (C-4'), 116.48 (C-5'), 122.16 (C-6'), Sugar moiety**:** 103.35 (C-1''), 74.04 (CH, C-2''), 77.16 (CH, C-3''), 70.11 (CH, C-4''), 76.37 (CH2, C-5''), 61,10(CH2, C-6'').

Compound 10 (Quercetin 3-*O*-β-xyloside): yellow powder, m.p. 235 ºC, soluble in methanol, positive Molisch's test (glycosidic nature), $\mathbf{R}_f$ = 0.45, (methylene chloride: methanol, 8:2), on silica gel sheets, dark yellow color with *p*-anisaldehde/ H_2SO_4 / heat at 110 ºC, yellow color in UV365 nm, yellow color with UV/NH_3 and the color is intensified with UV/$AlCl_3$. LC-HRMS $[M+H]^+$ m/z: 435.0919, MW: 434.08463, R*t*: 11.67 calculated for $C_{20}H_{18}O_{11}$. ^{1}H-NMR (400 MHz, CD_3OD): Aglycone part: δ 6.21 (1H, *d, J*=2.0, H-6), 6.40 (1H, *d, J*=2.1, H-8), 6.87 (1H, *d, J*=8.4, H-5'), 7.58 (1H, *dd, J*=1.8, 8.4 H-6'), 7.76 (1H, *d, J*=1.6, H-2'), Sugar moiety: 5.15 (1H, *d, J*=7.2 Hz, H-1''), 3.78 (1H, *dd, J*=11.4; 5.0 Hz, H-5''b), 3.46-3.54 (2H, *m*, H-3'', H-4''), 3.39 (1H, *m*, H-2''), 3.1 (1H, *dd, J*=11.5; 9.5Hz, H-5''a). DEPT-Q (100 MHz, CD_3OD): Aglycone part: δ δ179.17 (C, C-4), 165.79 (C, C-7), 162.41 (C, C-5), 157.54 (C, C-2), 156.96 (C, C-9), 149.85 (C, C-3'), 145.62 (C, C-4'), 134.80 (C, C-3), 123.15 (CH, C-6'), 122.89 (C, C-1'), 116.87 (CH, C-5'), 115.21 (CH, C-2'), 104.38 (C, C-10), 99.58 (CH, C-6), 94.72 (CH, C-8), Sugar moiety: 104.13 (CH, C-1''), 77.35 (CH, C-3''), 75.38 (CH, C-2''), 71.16 (CH, C-4''), 66.87 (CH2, C-5'').

Compound 11 (*p*-hydroxybenzoic acid): white needle crystals, m.p. 150 ºC, soluble in methanol, R_f = 0.35 in system [Methylene chloride: Methanol (90: 10 v/v)] and R_f = 0.17 in system [n-Hexane: Ethyl acetate (70: 30 v/v)], Blue fluorescence in $UV_{365\ nm}$ and a black spot at a wavelength of 254 nm, while give no color after spraying with p-anisaldehyde. LC-HRMS $[M-H]^-$ m/z: 137.02452, MW: 138.0318, R*t*: 8.57 calculated for $C_7H_6O_3$. ^{1}H-NMR (400 MHz, CD_3OD): δ 9.71 (1H, *s*, H of OH), 7.89 (2H, *d, J*= 8.7 Hz, H-2 and H-6), 6.83 (2H, *d, J*= 8.7 Hz, H-3 and H-5). DEPT-Q (100 MHz, CD_3OD): δ 168.47 (C-7), 162.16 (C-4), 130.99 (C-2 and C-6), 120.98 (C-1), 114.70 (C-3 and C-5).

Compound 12 (*p*-methoxybenzoic acid (*p*-anisic acid)): white needle crystals, m.p. 184 ºC, soluble in methanol, R_f = 0.83 in system [Methylene chloride: Methanol (90: 10 v/v)], Blue fluorescence in $UV_{365\ nm}$ and a black spot at a wavelength of 254 nm, while give no color after spraying with *p*-anisaldehyde, LC-HRMS $[M-H]^-$ m/z: 151.1463, MW:152.1472 R*t*: 1.63 calculated for $C_8H_8O_3$. ^{1}H-NMR (400 MHz, CD_3OD): δ 7.89 (2H, *d, J*= 8.7 Hz, H-2 and H-6), 6.83 (2H, *d, J*= 8.7 Hz, H-3 and H-5), 3.90 (3H, *s*, 3H of OCH_3). DEPT-Q (100 MHz, CD_3OD): δ168.83 (C-7), 161.94 (C-4), 131.65 (C-2 and C-6), 121.34 (C-1), 114.68 (C-3 and C-5), 55.0 (C-4).

Compound 13 (3,4- dihydroxybenzoic acid): white needle crystals, m.p. 179-182°C, soluble in methanol, R_f = 0.49 in system [Methylene chloride: Methanol (90: 10 v/v)], TLC investigation revealed invisible spot but showed a dark purple fluorescence under UV light which gave light brown with NH_3 and deep blue with $FeCl_3$ but no color after spraying with p-anisaldehyde. LC-HRMS $[M-H]^-$ m/z: 153.01943, MW: 154.0267, R*t*: 7.0 calculated for $C_7H_6O_4$. ^{1}H-NMR (400 MHz, CD_3OD): δ7.46 (1H, *dd, J*= 8.0, 2.0 Hz, H-6), 7.42 (1H, *d, J*= 2.0 Hz, H-2), 6.79 (1H, *d, J*= 8.0 Hz, H-5). DEPT-Q (100 MHz, CD_3OD): δ121.7 (C-1), 116.4 (C-2), 150.1 (C-3), 144.6 (C-4), 114.5 (C-5), 122.7 (C-6), 169.1 (C-7).

Compound 14 (*p*-coumaric acid): white needle crystals, m.p. 145-148°C, soluble in methanol, R_f = 0.45 in system [Methylene chloride: Methanol (90: 10 v/v)], TLC investigation revealed fant purple spot which showed a dark purple fluorescence under UV light at 254 nm but no color after spraying with *p*-anisaldehyde. LC-HRMS $[M-H]^-$ m/z: 163.0421, MW: 164.04784, R*t*: 10.97 calculated for $C_9H_8O_3$. ^{1}H-NMR (400 MHz, CD_3OD): δ 7.58 (1H, *d, J*=16.0 Hz, H-8), 7.45 (1H, *d, J*=8.4 Hz, H-2 and H-6), 6.81 (2H, *d, J*=8.4 Hz, H-3 and H-5), 6.29 (1H, *d, J*=16.0 Hz, H-7). DEPT-Q (100 MHz, CD_3OD): δ 170.05 (C-9), 159.64 (C-4), 144.90 (C-8) 131.57 (C-2 and C-6), 125.94 (C-1), 115.55 (C-3 and C-5), 114.60 (C-7).

Compound 15 (Quercetin-3-*O*-rutinoside (rutin)): yellow powder, m.p. 240-242 ºC, soluble in methanol, positive Molisch's test (glycosidic nature), R_f = 0.42, (ethyl acetate: methanol, 8:2), on silica gel sheets, dark yellow color with p-anisaldehde/ H_2SO_4 / heat at 110 ºC, yellow color in UV365 nm, yellow color with UV/NH_3 and the color is intensified with UV/$AlCl_3$. LC-HRMS $[M+H]^+$ m/z: 611.15994, MW: 610.1526, R*t*: 8.69 calculated for $C_{27}H_{30}O_{16}$. ^{1}H-NMR (400 MHz, CD_3OD): Aglycone part: δ7.89 (1H, *d, J*=2.0 Hz, H-2'), 7.57 (1H, *dd, J*=8.5, 2.0 Hz, H-6'), 6.87 (1H, *d, J*=8.5 Hz, H-5'), 6.35 (1H, *d, J*=2.0 Hz, H-8), 6.19 (1H, *d, J*=2,0 Hz, H-6), Sugar moiety: δ 5.02 (1H, *d, J*=7.8 Hz, H-1''), 4.55 (1H, *d, J*=1.8 Hz, H-1'''), 3.81 (1H, *dt, J*=10.9, 1.0 Hz, Hb-6''), 3.63 (1H, *dd, J*=3.5, 1.8 Hz, H-2'''), 3.54 (1H, *dd, J*=9.5, 3.5 Hz, H-3'''), 3.26-3.48 (4H, *m*, H-2'', H-3'', H-4'', H-5''), 3.44 (1H, *m*, H-5'''), 3.39 (1H, *m*, Ha-6''), 3.27 (1H, *m*, H-4'''), 1.14 (3H, *d, J*=6.1 Hz, H-6'''). DEPT-Q (100 MHz, CD_3OD): Aglycone part: δ 158.70 (C-2), 135.28 (C-3), 179.41 (C-4), 163.37 (C-5), 99.52 (C-6), 166.68 (C-7), 94.46 (C-8), 158.32 (C-9), 105.82 (C-10), 122.65 (C-1'), 117.21 (C-2'), 145.66 (C-3'), 115.56 (C-5'), 149.95 (C-4'), 122.93 (C-6'), Sugar moiety: δ 104.05 (C-1''), 75.05 (C-2''), 76.93 (C-3''), 71.19 (C-4''), 76.49 (C-5''), 67.43 (C-6''), 101.98 (C-1'''), 71.06 (C-2'''), 72.05 (C-3'''), 73.51 (C-4'''), 69.20 (C-5'''), 17.59 (C-6''').

Compound 16 (Luteolin-7-*O*-β-glucoside): faint yellow powder, m.p. 238-240 ºC, sparingly soluble in methanol, positive Molisch's test (glyosidic nature), R*f* = 0.42, S (chloroform: methanol, 8:2), on silica gel sheets, yellow color with *p*-anisaldehde/ H_2SO_4 / heat at 110 ºC, purple color in UV365 nm, yellow color with UV/NH_3 and the color is intensified with UV/$AlCl_3$, LC-HRMS [M+H] + m/z: 449.10745, MW: 448.10017, Rt: 10.41 calculated for $C_{21}H_{20}O_{11}$. ^{1}H-NMR (400 MHz, CD_3OD): A glycone part: δ 7.44 (1H, *dd, J*=8.4, 2.0 Hz, H-6'), 7.42 (1H, *d, J*=2.4 Hz, H-2'). 6.90 (1H, *d, J*=8.4 Hz, H-5'), 6.80 (1H, *d, J*=2.0 Hz, H-8), 6.75 (1H, *s*, H-3), 6.45 (1H, *d, J*=2.4 Hz, H-6), Sugar moiety: δ 5.07 (1H, *d, J*=7.6 Hz, H-1''). DEPT-Q (100 MHz, CD_3OD): A glycone part: δ182.4 (C-4), 164.9 (C-7), 163.4 (C-2), 161.5 (C-5), 157.4 (C-9), 150.4 (C-4'), 146.2 (C-3'), 121.8 (C-1'), 119.6 (C-6'), 116.4 (C-5'), 113.9 (C-2'), 105.7 (C-10), 103.6 (C-3), 100.0 (C-6), 95.2(C-8), Sugar moiety: δ 100.3 (C-1''), 77.6 (C-5''), 76.8 (C-3''), 73.5 (C-2''), 70.00 (C-4''), 61.07 (C-6'').

2. Docking study

Table S1. showed the distance and the interaction type between the most active metabolites (compound **1** and **3**) and the referenced drug (ribociclib) with the CDK-2 target enzyme.

Compound	Cyclin-Dependent Kinase 2 [CDK-2 (PDB ID: 1DI8)]			
	Affinity Kcal/mol	Distance (in Å) from main residue		Interaction
**Compound 1**	-13.2740	2.32	Asp145	H-Donor
		2.96	Lys33	H-Donor
		4.21	Val18	Hydrophobic
		2.08	Ile10	Hydrophobic
**Compound 3**	-13.4407	3.14	Asp145	H-Donor
		3.11	Phe80	H-Donor
		3.81	Lys129	Hydrophobic
		2.17	Val18	Hydrophobic
		2.19	Ile10	Hydrophobic
		3.59	Leu134	Hydrophobic
		4.78	Ala144	Hydrophobic
		4.25	Ala131	Hydrophobic
		4.69	Val64	Hydrophobic
**Ribociclib**	-17.5842	3.77	Asp145	H-Acceptor
		2.61	Lys33	H-Acceptor
		2.96	Lys89	H-Acceptor
		2.82	Ile10	Hydrophobic
		3.68	Leu83	Hydrophobic
		3.15	Phe80	Hydrophobic
		3.73	Phe82	Hydrophobic
		3.27	Val18	Hydrophobic
		4.00	Leu148	Hydrophobic
		3.17	Leu134	Hydrophobic
		3.79	Val64	Hydrophobic

Table S2. showed the distance and the interactions type between the most active metabolite (quercetin 3-xyloside) and the referenced drug (ribociclib) with the CDK-6 target enzyme.

Compound	Human Cyclin-Dependent Kinase 6 [CDK-6 (PDB ID: 1XO2)]			
	Affinity Kcal/mol	Distance (in Å) from main residue		Interaction
**Compound 10**	-16.2320	2.80	Gln149	H-Acceptor
		3.55	Gln149	H-Donor
		2.99	Asp163	H-Donor
		3.70	Asp163	H-Donor
		3.15	Asp102	H-Donor
		3.65	Ala 104	H-Donor
		2.81	Ile19	Hydrophobic
		3.35	Ala41	Hydrophobic Hydrophobic
		3.00	Val27	Hydrophobic
		2.92	Ala162	Hydrophobic
		2.79	Val77	
**Ribociclib**	-15.0820	3.18	Gln149	
		3.24	Ile19	H-Donor
		3.40	Asp163	H-Donor
		3.82	Ala141	H-Acceptor
		3.71	Leu152	Hydrophobic
		3.32	Phe98	Hydrophobic Hydrophobic
		3.04	Val27	Hydrophobic
		3.10	Ala162	Hydrophobic Hydrophobic
		3.85	Glu61	

Table S3. showed the distance and the interaction type between the most active metabolite (quercetin 3-xyloside) and the referenced drug (sorafenib) with the VEGFR-2 target enzyme.

Compound	Vascular Endothelial Growth Factor Receptor 2 [VEGFR-2 (PDB ID: 2OH4)]			
	Affinity Kcal/mol	Distance (in Å) from main residue		Interaction
**Compund 10**	-10.3913	3.02	Glu883	H-Donor
		3.10	Asp1044	Hydrophobic
		2.60	Arg1025	H-Acceptor
		3.02	His1024	Hydrophobic
		2.99	Ile1023	Hydrophobic
**Sorafenib**	-12.5811	2.83	Glu883	H-Donor
		2.78	Glu883	H-Donor
		2.67	Asp1044	H-Acceptor
		3.03	Cys917	H-Acceptor
		3.12	Cys1043	Halogen
		3.24	Ile1042	Halogen

Table S4. showed the distance and the interaction type between the most active metabolite (luteolin-7-O-glucoside) and the referenced drug (irinotecan) with the Topoisomerase I target enzyme.

Compound	Human DNA Topoisomerase I (PDB ID: 1T8I)			
	Affinity (Kcal/mol)	Distance (in Å) from main residue		Interaction
**Cpmpound 16**	-13.8309	2.94	Lys532	H-Acceptor
		2.76	Glu356	H-Donor
		3.03	DG12	H-Donor
		3.01	ASP533	H-Donor
		3.21	DT10	Hydrophobic
		3.38	DC112	Hydrophobic
		3.12	DA113	Hydrophobic
		3.14	TGP11	Hydrophobic
**Irinotecan**	-14.6530	3.21	Glu356	H-Acceptor
		2.95	ASP533DA113	Hydrophobic
		2.94	DC112	Hydrophobic
		2.60	Ile355	Hydrophobic
		3.01	His632	Hydrophobic
		2.83	Thr 718	Hydrophobic
		2.90		H-Donor

Table S5. The ADMET analysis of compounds **2,4–9, 11** and **12**.

Property	Model Name	Predicted Value									Unit
		Cpd 2	Cpd 4	Cpd 5	Cpd 6	Cpd 7	Cpd 8	Cpd 9	Cpd 11	Cpd 12	
Absorption	Water solubility	-3.072	-3.004	-3.025	-4.741	-3.372	-3.094	-3.011	-1.632	-1.865	Numeric (log mol/L)
	Caco2 permeability	1.171	1.429	1.539	0.472	0.587	0.096	-0.689	1.13	1.195	Numeric (log Papp in 10^{-6} cm/s)
	Intestinal absorption (human)	100	78.127	78.138	79.677	74.994	81.13	40.086	74.377	100	Numeric (% Absorbed)
	Skin Permeability	-2.735	-2.735	-2.745	-2.748	-2.735	-2.735	-2.735	-2.392	-2.576	Numeric (log Kp)
	P-glycoprotein substrate	No	Yes	Yes	Yes	Yes	Yes	Yes	No	No	Categorical (Yes/No)
	P-glycoprotein I inhibitor	No	No	No	Yes	No	No	No	No	No	Categorical (Yes/No)
	P-glycoprotein II inhibitor	No	No	No	Yes	No	No	No	No	No	Categorical (Yes/No)
Distribution	VDss (human)	-1.088	-1.266	-1.066	-1.163	0.239	1.153	0.241	-0.584	-1.856	Numeric (log L/kg)
	Fraction unbound (human)	0	0	0	0.078	0.015	0.168	0.112	0.479	0.454	Numeric (Fu)
	BBB permeability	-0.141	-0.821	-0.154	-0.785	-1.355	-0.907	-2	-0.331	-0.255	Numeric (log BB)
	CNS permeability	-1.187	-2.339	-1.178	-3.021	-3.432	-2.251	-4.982	-2.91	-2.236	Numeric (log PS)
Metabolism	CYP2D6 substrate	No	No	No	No	No	No	No	No	No	Categorical (Yes/No)
	CYP1A2 inhibitior	No	No	No	No	Yes	Yes	No	No	No	Categorical (Yes/No)
	CYP2C19 inhibitior	No	No	No	No	No	No	No	No	No	Categorical (Yes/No)

	CYP2C9 inhibitior	No	No	No	No	No	Yes	No	No	No	Categorical (Yes/No)
	CYP2D6 inhibitior	No	No	No	No	No	No	No	No	No	Categorical (Yes/No)
	CYP3A4 inhibitior	No	No	No	No	Yes	No	No	No	No	Categorical (Yes/No)
Excretion	Total Clearance	0.083	-0.34	-0.074	0.689	0.578	0.495	0.599	0.666	0.657	Numeric (log ml/min/kg)
	Renal OCT2 substrate	No	No	No	No	No	No	No	No	No	Categorical (Yes/No)
Toxicity	AMES toxicity	No	No	No	No	No	No	No	No	No	Categorical (Yes/No)
	Max. tolerated dose (human)	0.199	0.401	0.211	-0.887	1.062	0.499	0.705	1.002	0.734	Numeric (log mg/kg/day)
	hERG I inhibitor	No	No	No	No	No	No	No	No	No	Categorical (Yes/No)
	hERG II inhibitor	No	No	No	No	No	No	Yes	No	No	Categorical (Yes/No)
	Oral Rat Acute Toxicity (LD50)	2.346	2.688	2.355	2.571	2.295	2.455	2.844	1.862	2.125	Numeric (mol/kg)
	Hepatotoxicity	No	No	No	No	No	No	No	No	No	Categorical (Yes/No)
	Skin Sensitisation	No	No	No	No	No	No	No	No	No	Categorical (Yes/No)
	T.Pyriformis toxicity	0.285	0.285	0.285	0.285	0.301	0.326	0.285	0.218	0.233	Numeric (log ug/L)
	Minnow toxicity	-0.787	-2.004	1.456	0.811	1.408	3.169	2.573	2.21	1.853	Numeric (log mM)

Table S6. The ADMET analysis of compounds **13–15**.

**Property**	**Model Name**	**Predicted Value**			
		**Cpd 13**	**Cpd 14**	**Cpd 15**	**Unit**
Absorption	Water solubility	-2.069	-1.839	-2.892	Numeric (log mol/L)
	Caco2 permeability	0.49	1.144	-0.753	Numeric (log Papp in 10^{-6} cm/s)
	Intestinal absorption (human)	71.174	91.673	28.202	Numeric (% Absorbed)
	Skin Permeability	-2.727	-2.366	-2.735	Numeric (log Kp)
	P-glycoprotein substrate	No	No	Yes	Categorical (Yes/No)
	P-glycoprotein I inhibitor	No	No	No	Categorical (Yes/No)
	P-glycoprotein II inhibitor	No	No	No	Categorical (Yes/No)
Distribution	VDss (human)	-1.298	-0.607	0.011	Numeric (log L/kg)
	Fraction unbound (human)	0.648	0.421	0.291	Numeric (Fu)
	BBB permeability	-0.683	-0.239	-2.076	Numeric (log BB)
	CNS permeability	-3.305	-2.413	-5.742	Numeric (log PS)
Metabolism	CYP2D6 substrate	No	No	No	Categorical (Yes/No)
	CYP1A2 inhibitior	No	No	No	Categorical (Yes/No)
	CYP2C19 inhibitior	No	No	No	Categorical (Yes/No)
	CYP2C9 inhibitior	No	No	No	Categorical (Yes/No)
	CYP2D6 inhibitior	No	No	No	Categorical (Yes/No)
	CYP3A4 inhibitior	No	No	No	Categorical (Yes/No)
Excretion	Total Clearance	0.551	0.696	-0.28	Numeric (log ml/min/kg)
	Renal OCT2 substrate	No	No	No	Categorical (Yes/No)
Toxicity	AMES toxicity	No	No	No	Categorical (Yes/No)

	Max. tolerated dose (human)	0.814	0.758	0.427	Numeric (log mg/kg/day)
	hERG I inhibitor	No	No	No	Categorical (Yes/No)
	hERG II inhibitor	No	No	Yes	Categorical (Yes/No)
	Oral Rat Acute Toxicity (LD50)	2.423	2.07	2.445	Numeric (mol/kg)
	Hepatotoxicity	No	No	No	Categorical (Yes/No)
	Skin Sensitisation	No	No	No	Categorical (Yes/No)
	T.Pyriformis toxicity	0.273	0.211	0.285	Numeric (log ug/L)
	Minnow toxicity	2.451	1.815	2.846	Numeric (log mM)
